# Calmette's Ophthalmic Reaction in Tuberculosis
*Read at the meeting of the Bristol Medico-Chirurgical Society, May 13th, 1908.


**Published:** 1908-06

**Authors:** J. M. Fortescue-Brickdale

**Affiliations:** Physician, Royal Hospital for Sick Children and Women, Bristol; Director, Public Health Laboratory, University College, Bristol


### CALMETTE'S OPHTHALMIC REACTION IN TUBERCULOSIS.*

BY

J. M. FORTESCUE-BRICKDALE, M.A., M.D. Oxon.,

*Physician, Royal Hospital for Sick Children and Women, Bristol; Director, Public Health Laboratory, University College, Bristol.*

ABOUT the middle of last year von Pirquet[1] made a communication to the Berlin Medical Society, in which he pointed out that when tuberculin was injected hypodermically three reactions

* Read at the meeting of the Bristol Medico-Chirurgical Society, May 13th, 1908.

occurred. (1) A general reaction characterised by pyrexia, (2) a local exacerbation in the tuberculous foci, and (3) an inflammatory hyperæmia along the track of the needle. The last gave rise to what is termed the "cuti-reaction," in which a 25 per cent. solution of Koch's "old" tuberculin was applied to the scarified skin, much after the manner of ordinary Jennerian vaccination. In September he published a list of 100 cases (in children), in all of which the positive reaction (*i.e.* the appearance of a papule at the site of vaccination) had been obtained, and in all of which an autopsy showed the presence of tuberculous lesions, with the exception of one case, in which, however, old pleural adhesions were found.

Mainini,[2] who has tried von Pirquet's reaction in 208 cases, finds that a positive result is more frequently obtained than in the case of Calmette's ophthalmic reaction. This he explains by supposing that healed tuberculous foci respond to the former and not to the latter method.

Von Pirquet,[3] in a later communication, gives details of 300 children, some tuberculous and some not. 88 per cent. of the obviously tuberculous gave positive reactions, the remainder being severe cases of meningitis, etc. Of 109 apparently non-tuberculous infants, only two gave a positive reaction, but among similar children, aged 8 to 14 years, 35 per cent. responded.

Feer, who has recently extended and confirmed von Pirquet's results, also notes that the test is not applicable to adults, as the percentage of positive reactions increases with the age of the patient. He and others also state that in severe tuberculous infections, shortly before death, no reaction can usually be obtained. The view of most observers is that von Pirquet's reaction is a useful test when positive in young children, but that its absence cannot be taken definitely to exclude tuberculosis, while its presence may only indicate a healed lesion.

Calmette's modification of von Pirquet's cutaneous reaction is now too well known to need detailed description. It has now been largely tested both on the Continent, in America, and in this country. Mr. Sydney Stephenson was, I believe, one of the first to publish his results in an English journal, his series of over

30 cases appearing in the *British Medical Journal* of October 19th, 1907.

At the conclusion of his paper he notes that, following Calmette's own directions, he used the specially prepared tuberculin which is sold for the purpose. This consists of a 1 per cent. solution (or probably a suspension) of the dead bodies or fragments of tubercle bacilli in distilled water. The bacilli are obtained from ordinary tuberculin by precipitation with alcohol.

Calmette considered that the glycerin or the carbolic used in preparing Koch's tuberculin would irritate the conjunctiva and cause errors in the result.

MacLennan,[4] with a view of testing this, made seventy observations on Calmette's tuberculin—twenty-five with Koch's old tuberculin, and ten with the new tuberculin. The last-named preparation in 1 per cent. solution was found to give doubtful and unsatisfactory results, but with the old tuberculin (1 per cent.) MacLennan found the results obtained were the same as those with Calmette's special preparation. This is apparently borne out by the experience of German authors, who have largely employed Koch's original preparation. MacLennan has also instilled 1 per cent. solutions of glycerin and carbolic acid into normal eyes without obtaining any reaction, and these percentages are probably the maxima for Koch's T.A.

It is hardly surprising that Koch's T.R. should fail to give conclusive reactions, seeing that it is a much milder preparation than the old tuberculin, and that when used hypodermically it produces only a general, and no local reaction.

After Calmette's original paper was published, several observers tried to improve on his method by classifying the reactions obtained according to their severity. Webster and Kilpatrick,[5] however, as the result of observations on 123 cases, came to the conclusion that no relationship could be shown to exist between the severity or duration of the reaction, and the extent or activity of the tuberculous process, as evidenced by the physical signs in the chest and the pyrexia. Smithies and Walker,[6] who tried the test on 242 individuals, came to a similar conclusion,

Gulland and Williamson,[7] who report 28 cases, whilst noting that the time of onset varies more than is usually supposed, could determine no relationship between the age and condition of the patient, the state of the lungs, and the activity of the process on the one hand, and the severity of the reaction on the other. Wolff-Eisner and Teichmann,[8] however, have attempted to show that the type of reaction may have some prognostic value. They regard a brisk reaction, reaching its height in from twenty to twenty-four hours, keeping high the second day, and subsiding finally at latest on the fourth, as indicating a favourable condition for cure. They state that this type occurs in early cases, and in those in the first and second stage, and indicates a vigorous reaction on the part of the organism against the disease.

On the other hand, Stephens[9] thought that cases in which the lesions were early or healed reacted sluggishly; sometimes there was considerable delay. He admits, however, that some advanced cases also showed a delayed reaction.

Heywood[10] remarks that in advanced cases a negative reaction generally means a hopeless prognosis.

Delayed reactions sometimes occur. Smithies and Walker observed 6 cases of very slight or delayed reaction in their series. Some of these cases, when again tested, gave a severe reaction. A similar incident was reported by Eyre, Wedd and Hertz,[11] in their series of 138 individuals. I shall discuss the general question of the significance of second reactions later on.

General symptoms, pyrexia, malaise, etc., are but seldom observed. One such case occurred at the Children's Hospital out of thirty-two individuals. Woodcock[12] observed pyrexia in 2 out of 200 cases, and many cases of headache without pyrexia. Audeoud,[13] contrary to most observers, believes that slight pyrexia (1° C.) is the rule. Eisen,[14] out of 82 cases, noted pyrexia in 2, which, curiously enough, were both negative reactions. Webster and Kilpatrick observed 7 cases of pyrexia in 123 patients submitted to the test.

Another curious occurrence which occasionally takes place is a recrudescence of the ophthalmia. One of the cases in the Children's Hospital developed a second attack of conjunctivitis seven weeks after the instillation, and five weeks after all signs of the first inflammation had passed off.

Long[15] points out that the reaction is occasionally bilateral. The reaction in the second eye was observed in 2 out of 40 cases, twenty-four and forty-eight hours respectively after the instillation. This phenomenon appears to be due to some accidental transference of the material from one eye to the other.

Calmette's reaction has been described as harmless and safe. This, however, is not absolutely the case. The literature shows that in a certain proportion of cases serious, or at least inconvenient, results occur. It is, of course, of importance to remember that any kind of conjunctivitis is a contra-indication for the test, as not only does it become difficult in some cases to determine whether a reaction has taken place or not, but in others the condition may be very seriously aggravated. Smithies and Walker, however, are of opinion that the instillation of Calmette's tuberculin does not of itself aggravate a pre-existing conjunctivitis.

Comby[16] holds that both eyes should be normal if the test is to be satisfactorily applied, but Brunetière[17] advises that in the case of disease in one eye, the other may safely be instilled with Calmette's preparation.

As to patients with definite disease of the eye, I have been able to collect the following observations from the literature.

Lapersonne[18] notes severe reaction in 6 cases; in all but one of these there was a pre-existing lesion. The cases included 2 of irido-cyclitis. In none was there any permanent impairment of vision.

Walker[19] reports 3 cases of ocular disease which reacted severely. In one of these a phlyctenular ulcer appeared on the cornea, which healed, however, without scarring.

Brunetière reports one case of phyctenular keratitis following Calmette's reaction, which he thinks may have been a recurrence, independent, more or less, of the tuberculin instillation.

Ramsay[20] reports a violent reaction leading to permanent corneal opacity in a patient who was obviously tuberculous.

At the time of instillation there was a slight nebula in the cornea of the eye, while the other eye had a superficial vascular ulcer, which healed up under injections of tuberculin.

Waldstein[21] states that a violent reaction may occur in follicular conjunctivitis, or even in ordinary catarrh.

Woodcock reports severe reactions in cases presenting ocular lesions, and Brons[22] has reported 5 similar cases.

Knapp[23] has noted interstitial keratitis after Calmette's reaction.

Even cases in which there has been ocular trouble in the past may give rise to severe reactions. Thus Eisen, out of 73 cases, noted 2 severe ones in which there was a history of ophthalmia in childhood. Wiens and Günther, among 47 cases, reported 3, in one of which there had been conjunctivitis, and Comby noted one similar instance. Kalt[24] reports on an old tuberculous lesion which was apparently lighted up again by tuberculin instillation.

On the other hand, Stephenson has reported on 30 cases of his own, and 21 of French authorities, without any mishap. Truc, the professor of ophthalmology at Montpelier, observed no bad results among 23 eye patients.

Reports on cases in which severe conjunctivitis appeared without any previous ocular lesion include 5 out of 30 examined by Collin;[25] in 5 or 6 noted by Butler[26] there was iritis and chororiditis; and Heywood noted one case in which the symptoms only slowly cleared up after six weeks.

Feer[27] considers that scrofulous children often react severely, and Delorme[28] has collected 39 cases of conjunctivitis from the literature. Napier[29] notes two curious cases in which, after Calmette's test had been applied, the injection of Koch's T.R. produced severe local reaction. MacLennan has also noted a tendency to recrudescence on injection with T.R. Epstein has reported 2 cases of phlyctenules, and one case of slight keratitis in children, and Mackay[30] an ophthalmia in a woman lasting ten weeks.

Though, no doubt, this does not by any means represent the total number of cases in which untoward results have taken place, yet, considering that the number of times in which Calmette's test has been made now reaches some tens of thousands, it would not appear unfair to conclude that such accidents as keratitis or phlyctenular ulcers are very rare, especially when both eyes are healthy, and there is no history of antecedent eye affections.

It was owing to the fact that in two cases a prolonged reaction (eight to ten days) occurred that Comby substituted a 5 per cent. for the 10 per cent. solution recommended by Calmette. Feer, however, regards a 5 per cent. solution of T.A. an uncertain re-agent, and has, moreover, observed one case of phlyctenular ulceration under its use. MacLennan takes the same view as Comby, but quotes Oliver and Terras[31] as stating that only 1 in 150 in adults of Calmette's tuberculin gives doubtful results. Heywood considers that 5 per cent. is in some cases too strong a solution for children.

Calmette's reaction, like that of von Pirquet, does not usually occur in very advanced cases. Comby has obtained a reaction in moribund cases, and also during all stages of such a severe infection as tuberculous meningitis. It differs from that of von Pirquet in not responding when absolutely quiescent foci of disease are present, and it is this which gives it its practical usefulness in adult cases. Patients probably susceptible to tuberculosis do not react. Calmette[32] obtained negative results with the infants of twelve tuberculous mothers.

Animals susceptible to tuberculosis apparently react very similarly to man. Thus Campbell and White,[33] using Koch's T.A. and not Calmette's preparation, found that among twenty cattle the results were the same as those obtained by tuberculin injections. The more advanced cases, however, reacted less markedly. Calmette's reaction appeared to have one advantage over tuberculin injections in cattle. The latter cannot be repeated, as subsequent injections are always negative, whereas Calmette's reaction can be taken after a tuberculin injection, and yet give a positive result. It may, therefore, be used to check fraudulent owners who inject their cows in order to protect them against any subsequent application of the tuberculin test.

Variations in the severity, and even in the occurrence of the reaction occur, and these have been explained as depending on a positive or negative phase in the individual. Eyre thinks that a negative phase, somewhat prolonged, occurs after an instillation. He has not, however, made any exact observations on this point.

As to the reliability of the test, it appears that while in certain cases it has been shown to be erroneous, in the majority it gives an accurate result.

In support of the first of these propositions may be quoted Serafini's[34] observation: 3 certain though mild cases of tuberculosis failed to react. Engel and Bauer[35] obtained a positive reaction in an infant under twelve months old, in which no signs of tuberculosis could be found (*post-mortem*), and the same result was obtained in 4 others who later on failed to react to a diagnostic injection of tuberculin. Chauffard[36] has recorded a case of chronic appendicitis in which a negative result was obtained, but which at a subsequent operation was shown to be tuberculous. Blum,[37] out of 5 negative cases, found tuberculous lesions in 4 (*post-mortem*).

Kraus, Lensenberger and Russ,[38] among 7 negatives, found no traces of tuberculosis in 2 (*post-mortem*), old apical scars in 2, and calcified bronchial glands in 1. Massary and Weil[39] have recorded a case of generalised carcinoma in which a positive reaction was obtained, but no traces of tubercle could be found at the autopsy.

Besides these cases, there are now many cases recorded of enteric fever, scarlet fever, etc., which have given positive reactions both in the pyrexial and apyrexial stages. The absence of an autopsy, however, deprives this evidence of some of its weight.

I have constructed a table from the literature, to which I have added the cases observed by my colleagues and myself at the Children's Hospital, the total number of individual cases reaching 4,478. The cases are arranged in three columns: those clinically tuberculous, those clinically non-tuberculous, and those clinically doubtful.

| Author. | Clinically Tuberculous | | Clinically non-Tuberculous. | | Clinically Doubtful. | |
|---|---|---|---|---|---|---|
| | + | – | + | – | + | – |
| Calmette and others .. .. | 63 | — | — | 52 | — | — |
| Grasset and Raimbault .. | 7 | 1 | 1 | 14 | 5 | 3 |
| Derchend .. .. .. | 7 | — | — | 13 | 1 | — |
| Prouff .. .. .. .. | 9 | — | — | 6 | 3 | — |
| Des Plats .. .. .. | 4 | — | — | 5 | — | — |
| Helipré and Hue .. .. | 14 | — | — | 8 | — | — |
| Grillot .. .. .. .. | 7 | — | — | 2 | — | — |
| Desbonnets .. .. .. | 2 | — | — | 2 | — | — |
| Letulle .. .. .. | 81 | 3 | — | 27 | — | — |
| Montegnon .. .. .. | 12 | 2 | — | 8 | 5 | — |
| Aubaret and Magne .. .. | 11 | — | — | — | 3 | 1 |
| Smithies and Walker .. | 176 | 9 | 2 | 186 | — | — |
| Mantoux .. .. .. | — | — | 16 | 184 | — | — |
| MacLennan .. .. .. | 54 | 5 | 16 | 9 | 9 | 3 |
| Kirkpatrick and Webster .. | 79 | 22 | — | 2 | — | 18 |
| Stephens .. .. .. | 88 | 16 | — | — | — | 2 |
| Woodcock .. .. .. | 5 | 2 | 3 | — | — | — |
| Parkes Weber .. .. | — | 5 | 5 | — | — | — |
| Mielle .. .. .. .. | 11 | — | — | 7 | — | — |
| Grünbaum and Austin .. | 18 | 2 | — | 52 | — | — |
| Eyre, Wedd and Hertz .. | 63 | 2 | — | 63 | — | 12 |
| Schröder and Kauffmann .. | 43 | 5 | 12 | 8 | 4 | 5 |
| Lenhartz .. .. .. | 33 | 4 | 4 | 7 | 24 | 40 |
| Cohn .. .. .. .. | 60 | 26 | 10 | 182 | 23 | 9 |
| Citron .. .. .. .. | 25 | 6 | 1 | 44 | 11 | 3 |
| Köhler .. .. .. .. | 161 | 8 | — | — | 5 | — |
| Eppenstein .. .. .. | 60 | 25 | 4 | 69 | 38 | 51 |
| Baldwin .. .. .. | 50 | 3 | 16 | 41 | 8 | 18 |
| Métraux .. .. .. | 14 | 1 | 2 | 40 | 9 | 2 |
| Schenk and Seyffert .. | 27 | 1 | 26 | 26 | 15 | 5 |
| Audeoud .. .. .. | 12 | 1 | — | 15 | 1 | 2 |
| Franke and Ratjen .. .. | 6 | — | 4 | 29 | 9 | 3 |
| Eisen .. .. .. .. | 23 | 22 | 5 | 23 | 4 | 5 |
| Comby .. .. .. .. | 10 | — | — | 8 | — | — |
| Necker and Paschkis .. | 42 | — | — | 22 | — | — |
| Plehn .. .. .. .. | — | — | 17 | 15 | — | — |
| Rosenan and Anderson .. | — | — | — | 12 | — | — |
| Levy .. .. .. .. | 34 | 7 | 6 | 235 | 32 | 22 |
| Zaniboni .. .. .. | 32 | — | — | 17 | — | — |
| Bandler and Kreiblich .. | 22 | 4 | 22 | 15 | — | — |
| Engel and Bawer .. .. | — | — | 5 | — | — | — |
| Mainini .. .. .. | 11 | 1 | 8 | 42 | 26 | 14 |
| Truc .. .. .. .. | 4 | — | — | — | — | 19 |
| Chauffard .. .. .. | — | 1 | — | — | — | — |
| Wiens and Günther .. .. | 6 | 5 | 7 | 26 | 2 | 4 |
| Klevienberg .. .. .. | 8 | 9 | — | — | — | — |
| Blum .. .. .. .. | — | — | 7 | 181 | 34 | 190 |
| Kraus and others .. .. | 21 | 5 | 11 | 1 | — | — |
| Bourget .. .. .. | — | — | 2 | — | — | — |
| Massary and Weil .. .. | — | — | 2 | — | — | — |
| Bristol Children's Hospital .. | 4 | 1 | — | 19 | 4 | 4 |
| Total .. .. .. | 1419 | 204 | 214 | 1931 | 275 | 435 |

Out of 1,623 cases in the first class, 1,419, or 87.4 per cent., gave a positive, and 204, or 12.6 per cent., a negative result. In the second class there are 1,931 cases, of which 214, or 11.1 per cent., were positive, and 1,717, or 88.9 per cent., negative. In the third class, out of 710 cases 275, or 38.7 per cent., were positive, and 435, or 61.3 per cent., were negative.

The figure 87.4 per cent. seems somewhat low for the positive results among clinically tuberculous cases. It must, however, be remembered that among the negative cases occur a certain number of cases of healed phthisis which are clinically counted as tuberculous, and also a considerable proportion of very severe or moribund cases, in which Calmette's reaction is usually, though not always, negative. If such were excluded, the proportion of successful results would be higher, and it is in the earlier cases that this test finds its sphere of usefulness clinically.

Smithies and Walker classify the possible sources of error under five headings: (1) Failure in technique; (2) Differences in the preparation of the tuberculin; (3) Clinical errors and difficulties in diagnosis; (4) Errors due to different views as to what constitutes a positive reaction to Calmette; (5) Our general ignorance as to possible modifying factors.

No doubt if these various sources of error could be eliminated, the percentage of accurate results reported would be higher.

The causation of Calmette's reaction, from the pathological point of view, is an interesting and somewhat obscure problem. It belongs to a class of phenomena which depend on the condition called in German *ueber-empfindlichkeit*, or over-sensitiveness. The Americans call it anaphylaxis; and in England, with our usual disregard of philology, we generally call it hypersensibility. The ordinary reactions to tuberculin and mallein are well-known examples of this condition, which is not confined, however, to bacterial proteins. A guinea-pig, for instance, injected with $\frac{1}{250}$ cc. of normal horse serum, is rendered so over-sensitive, that after an incubation period of ten to twelve days a second injection is usually fatal. The reaction sometimes observed in man after the injection of diphtheria antitoxin has a similar incubation period, and is probably of a like nature.

These various reactions are all specific, and are followed by a species of immunity, called by von Pirquet " allergie."

With regard to Calmette's reaction, Wassermann explains it by saying that it depends on the fact that the tissue cells, which have under the influence of the infection developed antibodies, can perform their function more rapidly and intensely under the influence of a very small dose of the poison, than cells which have never been called upon to react in this way at all. They are thus in a condition of over-sensibility towards the antigen.

That some local condition of over-sensitiveness is produced is shown by the fact that a second instillation of tuberculin into the conjunctival sac is in most cases positive ; at any rate, it is so frequently positive, even after the first injection has been negative, as to give it no clinical value. Smithies and Walker think that the soluble toxins absorbed by the conjunctival vessels give rise to the local production of tuberculo-tropic substances such as opsonins and lysins, which in their turn act on the bacillary bodies lying enmeshed in the tissues. These are then broken up, and irritating endotoxins are liberated which cause an inflammatory reaction. In non-tuberculous individuals the cells are not sensitive enough to respond with lysin or opsonin formation on absorbing the soluble toxins. The only thing that strikes one about this explanation is that it sounds almost too ingenious.

For practical clinicians the moral of Calmette's reaction is that there is no short cut to diagnosis, unless it be that made by the surgeon's knife when he opens the abdomen, for instance, and finds acute hemorrhagic pancreatitis.

### REFERENCES.

(1) **Pirquet**—*Wien. med. Wchnschr.*, 1907, lvii. 1369.
(2) **Mainini**—*Münch. med. Wchnschr.*, 1907, liv. 2583.
(3) **Pirquet** quot. Mainini.
(4) **MacLennan**—*Brit. M. J.*, 1907, ii. 1642.
(5) **Webster and Kilpatrick**—*Ibid.*, p. 1644.
(6) **Smithies and Walker**—*J. Am. M. Ass.*, 1908, l. 259.
(7) **Gulland and Williamson**—*Scottish M. & S. J.*, 1908, xxii. 317.
(8) **Wolff-Eisner and Teichmann**—*Berl. klin. Wchnschr.*, 1908, xlv. 65.

(9) **Stephens**—*Brit. M. J.*, 1908, i. 742.

(10) **Heywood**—*Brit. M. J.*, 1908, i. 626.

(11) **Eyre, Wedd and Hertz**—*Lancet*, 1907, ii. 1752.

(12) **Woodcock**—*Brit. M. J.*, 1908, i. 742.

(13) **Audeoud**—*Rev. méd. de la Suisse Rom.*, October, 1907.

(14) **Eisen**—*Beit. z. Klinik der Tuberk.*, 1907, viii., heft 4.

(15) **Long**—*Brit. M. J.*, 1907, ii. 1826.

(16) **Comby**—*Bull. méd.*, November 20th, 1907.

(17) **Brunetière**—*Gaz. Hebd des Sc. Méd. de Bordeaux*, 1907, xxviii. 615.

(18) **Lapersonne**—*Presse méd.*, 1907, xv., No. 99.

(19) **Walker**—*Lancet*, 1908, i. 640.

(20) **Ramsay**—*Lancet*, 1908, i. 716.

(21) **Waldstein** quot. Parkes Weber—*Brit. M. J.*, 1908, i. 386.

(22) **Brons** quot. Butler, *loc. cit.* — *Klin. Monart. f. Augenheilk.* January, 1908.

(23) **Knapp** quot. Butler.

(24) **Kalt** quot. Butler—*Rec. d'Ophtal.*, October, 1907, p. 595.

(25) **Collin**—*Med. Klin.*, January 26th, 1908.

(26) **Butler**—*Brit. M. J.*, 1908, i. 922.

(27) **Feer**—*Münch. med. Wchnschr.*, 1908, lv. 6.

(28) **Delorme**—*J. Am. M. Ass.*, 1908, l. 733.

(29) **Napier**—*Glasgow M. J.*, 1908, lxix. 4.

(30) **Mackay**—*Boston M. & S. J.*, 1908, clviii. 352.

(31) **Oliver and Terras**—*Presse méd.*, 1907, xv. 393.

(32) **Calmette**—*Bull. de Acad. de Méd.*, 1908, lix. 98.

(33) **Mc.Campbell and White**—*J. Exp. Med.*, 1908, x. 232.

(34) **Serafini**—*Giorn. del R. Accad. di Med. Torino*, November, 1907.

(35) **Engel and Bauer** quot. Mainini, *loc. cit.*

(36) **Chauffard**—*J. Am. M. Ass.*, 1908, l. 57.

(37) **Blum**—*Münch. med. Wchnschr.*, 1908, lv. 60.

(38) **Kraus, &c.**—*Wien. klin. Wchnschr.*, 1907, xx. 1385.

(39) **Massary and Weil**—*Bull. de la Soc. Méd des Hôp.*, November, 1907.

For the statistical table the references in some cases are the same as those quoted in the text. The rest were obtained as follows : Calmette, Grasset and Raimbault, Derchand, Prouff, Des Plats, Helipré and Hue, Desbonnets, Montegnon, Aubaret and Magne, from Smithies and Walker ; Letulle and Mantoux from MacLennan ; Parkes Weber, *Brit. M. J.*, i. 386, 536, 1908 ; Meille, *Brit. M. J.*. 1908, i. Epitome, p. 5 ; Grünbaum and Austin, *Proc. Roy. Soc. Med.*. 1907-08, i. Path. Sect., 74 ; Necker and Paschkis, *Wien. klin. Wchnschr.*, 1908, xxi. 316 ; Plehn, *Deut. med. Wchnschr.*, February 20th, 1908 ; Rosenau and Anderson, *J. Am. M. Ass.*, 1908, l. 961, ; Levy, *Deut. med. Wchnschr.*, January 16th, 1908, xxxiv. ; Zaniboni, *Il Policlinico.*, 1908, xv., No. 1 ; Bandler and Kreiblich quot. Mainini, *loc. cit.* ; Engel and Bauer, *ibid.* ; Truc quot. by Lapersonne ; Bourget, *Rev. méd. de la Suisse Rom.*, 1907, p. 888. The remainder are from the table by Schröder and Kaufmann, *Münch. med. Wchnschr.*, January 14th, 1908.

